# The unappreciated role of developing B cells in chronic gammaherpesvirus infections

**DOI:** 10.1371/journal.ppat.1012445

**Published:** 2024-09-19

**Authors:** Yiping Wang, April Feswick, Vasiliki Apostolou, Scott A. Tibbetts

**Affiliations:** 1 Department of Preventive Veterinary Medicine, Research Center for Swine Diseases, College of Veterinary Medicine, Sichuan Agricultural University, Chengdu, Sichuan, China; 2 Department of Molecular Genetics and Microbiology, UF Health Cancer Center, UF Genetics Institute, College of Medicine, University of Florida, Gainesville, Florida, United States of America; Mount Sinai School of Medicine, UNITED STATES OF AMERICA

## Abstract

The oncogenic gammaherpesviruses, including human Epstein–Barr virus (EBV; HHV-4), human Kaposi’s sarcoma-associated herpesvirus (KSHV; HHV-8), and murine gammaherpesvirus 68 (MHV68; MuHV-4; γHV68), establish lifelong latency in circulating B cells and directly contribute to the genesis of numerous types of malignancies including B cell lymphomas. Mounting evidence also implicates these viruses in autoimmune diseases such as multiple sclerosis. The prevailing paradigm for gammaherpesvirus biology holds that these viruses initially infect naïve B cells, then drive infected B cells independent of antigen stimulation through germinal center reactions and into the memory B cell compartment, which serves as a stable latency reservoir. However, cumulative findings from both humans and mice provide provocative evidence that suggests that B cells may play a central role in the natural lifestyle of gammaherpesviruses even before these cells reach full maturity. Here, we review the recent advancements in our understanding of the roles of developing B cells during gammaherpesvirus infection and disease and propose a new working model for latency establishment that incorporates developing B cell infection into the textbook paradigm.

## Introduction

The human gammaherpesviruses Epstein–Barr virus (EBV; HHV-4) and Kaposi’s sarcoma-associated herpesvirus (KSHV; HHV-8) are ubiquitous pathogens that are directly associated with the development of numerous cancer types, including B cell lymphomas [1–5]. In recent years, the long held postulate that these viruses may be associated with the genesis of autoimmune diseases such as multiple sclerosis (MS) has also gained new traction [6]. The primary reservoir for lifelong virus latency is the memory B cell compartment. Though it is clear that gammaherpesviruses have evolved numerous mechanisms to manipulate B cell signaling pathways and evade host immune surveillance [1–3,7,8], the specific mechanisms by which they establish and maintain latency in circulating mature B cells in vivo are not completely understood.

The predominantly held paradigm holds that gammaherpesviruses initially infect mature but naïve B cells, and in turn provide intrinsic signals that drive these cells, even in the absence of antigen stimulation, to enter germinal centers [5,9–11]. There, they proliferate and expand before exiting as resting memory B cells which harbor latent virus genomes, ostensibly for life. Certainly, empirical evidence supports many aspects of this concept. However, cumulative findings from both humans and mice suggest that there may be an important prequel to this story that has yet to be fully revealed: early and ongoing infection of B cells prior to their full maturation and entry into circulation. Here, we examine current evidence and posit the potential key role that developing B cells may play in chronic gammaherpesvirus infections.

## Developing B cells are targets of gammaherpesvirus infection

A growing body of circumstantial evidence from humans and empirical data from mice suggests that gammaherpesviruses may infect some B cells prior to their maturation. B cells differentiate from hematopoietic precursor cells in the bone marrow (BM), where they progress through multiple developmental stages including pro-B, pre-B, and immature B cells [12]. Immature B cells then enter circulation as transitional B cells before completing their maturation to become naïve follicular B cells or marginal zone B cells [12]. Whether gammaherpesviruses infect developing B cells during a natural course of infection in healthy people has not been determined. However, in the context of disease, numerous individual case studies have implicated the BM and progenitor B cells as potential sites of latent EBV or KSHV infection. Both EBV and KSHV have been detected in the BM of AIDS patients [13,14]. EBV is also found in BM biopsies of patients with hemophagocytic lymphohistiocytosis, a rare but lethal BM hemophagocytosis caused by EBV infection [15,16]. In the context of patients with X-linked lymphoproliferative diseases that lack conventional memory B cells, EBV is found in transitional (CD27-CD10+) B cell subsets [17]. Additionally, KSHV has been detected in immature B cells in the BM of transplant patients and in circulating human CD34+ hematopoietic progenitor cells of Kaposi’s sarcoma patients [18,19]. Though these individual findings have been largely overlooked, this cumulative evidence demonstrates that EBV and KSHV hold the capacity to infect both the site of primary hematopoiesis and developing B cells themselves.

In vitro studies have also demonstrated that human gammaherpesviruses are able to infect multiple subtypes of developing B cells. For example, EBV can infect and transform primary pro-B, pre-B, and immature B cells from fetal BM and fetal liver [20–22], and KSHV infects a human B cell line which displays characteristics (CD27-CD10+) of transitional B cells [23]. Interestingly, Sjögren’s syndrome patients with a recent EBV infection or EBV reactivation display increased numbers of transitional B cells as compared to patients with no recent EBV infection [24], suggesting that EBV may directly promote transitional B cell proliferation. Moreover, KSHV was shown to ex vivo infect primary transitional B cells isolated from human tonsil lymphocytes [25].

Further support for this hypothesis comes from murine studies, which allow for the assessment of developing B cell infection during a full course of natural infection. Murine gammaherpesvirus 68 (MHV68; MuHV-4; γHV68) is a pathogen of murid rodents that is genetically related to EBV and KSHV. MHV68 mirrors several key features of human gammaherpesvirus pathobiology, including the establishment of lifelong latency in B cells [7,8], and therefore offers the opportunity to systematically study key facets of in vivo infection. It is thus notable that following intranasal inoculation of wild-type mice, MHV68 is detectable in the BM as early as 5 days and is present throughout chronic infection [26–32]. Moreover, in chronically infected mice, MHV68 is detectable in pro-B/pre-B cells and immature B cells in the BM, as well as transitional B cells in the spleen [32], demonstrating that infection of the developing B cell compartment is a normal feature of long-term infection. These findings, along with evidence from EBV and KSHV studies, demonstrate that gammaherpesviruses hold an inherent capacity to infect developing B cells, and thus bring forth the intriguing possibility that human disease-based case studies may simply reflect the reality that the BM is a reservoir for EBV and KSHV during normal infection.

The observation that virus is detectable in immature and transitional B cells at stable levels during chronic infection is particularly remarkable because these cells have a lifespan of only 24 to 72 h, suggesting that there is a source of virus that is available in the BM for constant, low-level, de novo infection of newly generated developing B cells. What is that source of virus? The answer to that question remains to be resolved, but the most likely suspect would seem to be plasma cells. It has been well established for EBV, KSHV, and MHV68 that differentiation of infected B cells into plasma cells triggers reactivation from latency [33–37], though de novo infection of plasma cells may result in latency in some scenarios [25]. Because long-lived plasma cells routinely transit to the BM (reviewed in [38–40]), it is plausible that these cells provide new virus particles for infection of developing B cells. It is also conceivable that another cell type within the BM acts as a supplier. The BM is a vastly complex organ, with primary hematopoiesis generating multiple cell lineages among a milieu of stromal cells and mature immune cells, and alongside osteoblasts, osteoclasts, and endothelial cells. For example, KSHV has shown the capacity to infect bone marrow stromal cells [41,42]. Additionally, KSHV has been demonstrated to infect endothelial cells derived from BM [43], although as of yet there is no direct evidence of BM endothelial cell infection. However, it remains to be determined whether any of these cell types could serve as a long-term reservoir to support infection of newly generated B cells.

## Developing B cells contribute to chronic infection and disease

In both humans and mice, gammaherpesvirus latency is sustained at a steady level over time, with a latency “set point” achieved after initial expansion of latently infected cells [44–46]. While it is clear that many B cell subtypes can support initial gammaherpesvirus infection and expansion, the potential connection between specific infected B cell subtypes and their subsequent roles in the maintenance of long-term latency are largely unknown. Nevertheless, studies using MHV68 have shed some light on the potential contribution of developing B cells to chronic infection. Most notably, depletion of IL-7 (a cytokine which is required for developing B cell survival) after latency is established impairs the maintenance of long-term infection in the mature B cell compartment [32]. This finding strongly suggests that recurrent infection of developing B cells may play a crucial role in maintaining the latency “set point” observed during lifelong infection in both mice and humans.

Although it has not yet been determined whether human gammaherpesvirus infection of developing B cells directly contributes to pathogenesis, some evidence supports the correlation of high numbers of circulating developing B cells with the development of EBV+ B cell malignances. It is widely believed that co-infection of malaria and EBV contributes to the high prevalence of Burkitt lymphoma in young children in malaria holoendemic regions of Africa [47]. Thus, it is notable that infants from a malaria- and Burkitt lymphoma-endemic region of western Kenya exhibit higher numbers of transitional B cells (CD10+CD34-), despite a normal distribution of naïve B cells (IgD+CD27-) and class-switched memory B cells (IgD-CD27+) [48], and also display higher EBV loads [49]. It is also notable that chronic HIV patients, who are at high risk for development of gammaherpesvirus malignancies, exhibit simultaneously increased frequencies of immature or transitional B cells and high EBV loads, even in situations in which memory B cells have been depleted [50]. Interestingly, the development of EBV-driven non-Hodgkin’s lymphoma in humanized mice correlates with the presence of a significant fraction of immature B cells at the time of inoculation, suggesting that EBV infection of developing B cells may contribute to this malignancy in this context [51].

To date, little is known about viral determinants of developing B cell infection. However, the use of virus gene editing technologies coupled with specific analysis of developing B cell populations may provide the opportunity for significant new insights. If indeed pre-B or other early-stage B cells are regularly infected, the implications for disease are significant. Specifically, the expression of virus gene products in cells prior to B cell selection processes could allow the survival of B cells that would undergo clonal deletion, perhaps leading to the survival of autoreactive B cells. Supporting this possibility, while deletion of the MHV68 vBcl-2 results in a modest reduction of total splenocyte infection [52–55], analysis of specific developing B cell subtypes revealed a critical role for vBcl-2 specifically in immature and transitional B cells, B cell subtypes that undergo selection and deletion prior to full maturation [32]. Similarly, in vitro KSHV infection of human tonsil samples with a mutant virus deficient in the highly conserved glycoprotein H (gH) resulted in more pronounced rates of infection in samples with high levels of transitional B cells [56], suggesting that KSHV infection of transitional B cells may partially rely on gH-dependent entry mechanisms. Thus, similar work with other gammaherpesvirus genes is warranted to determine whether EBV-, KSHV-, and MHV68-encoded genes have conserved functions that are of particular importance in developing B cells.

## A new working model for chronic gammaherpesvirus infection

**Fig 1:**
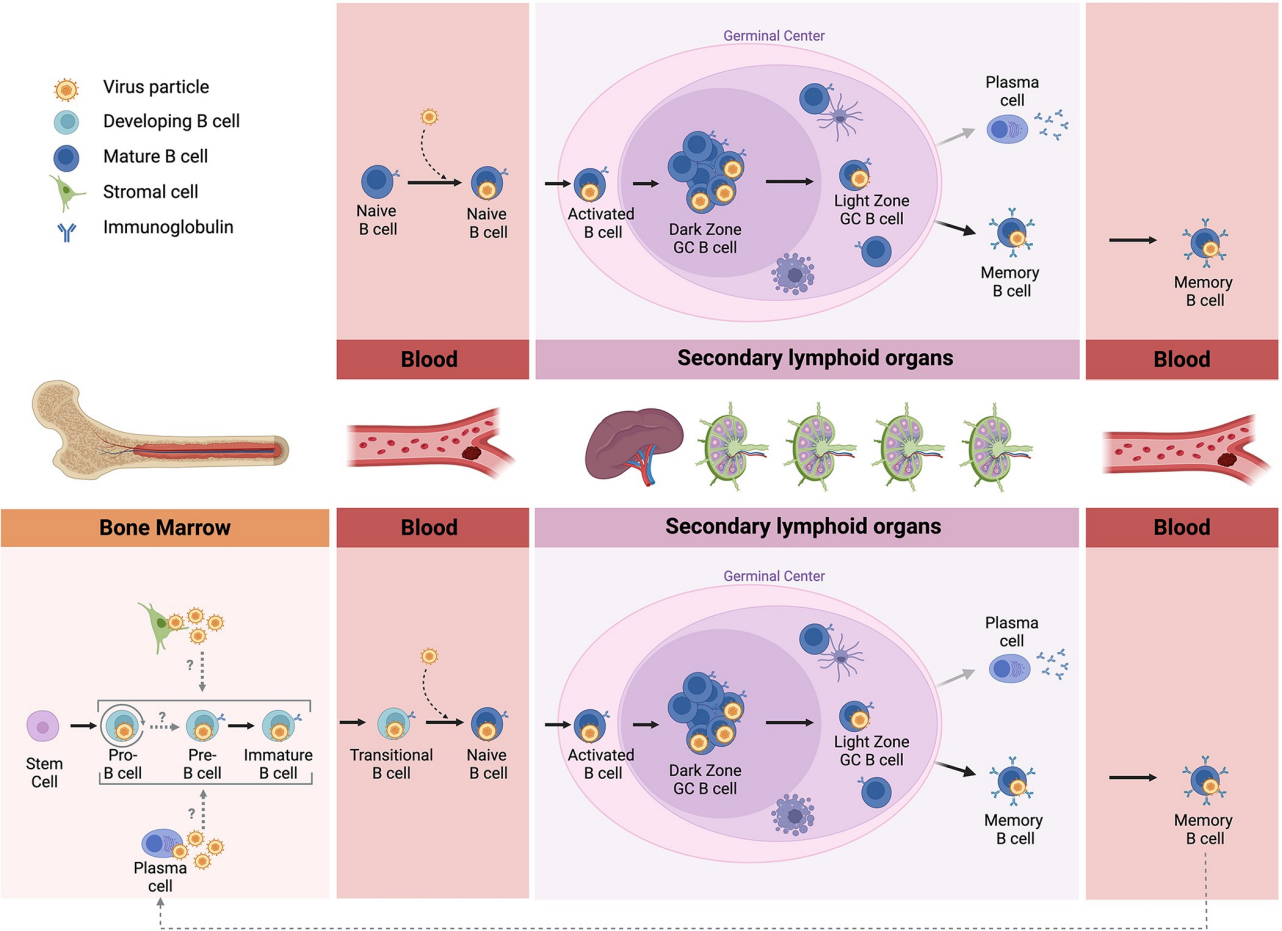
Models of gammaherpesvirus B cell infection dynamics. (Top) The prevalent paradigm holds that gammaherpesviruses infect naïve B cells and, independent of antigen, drive infected cells through the germinal center reaction and into the memory B cell compartment, which serves as the lifelong latent reservoir for virus. (Bottom) Working model incorporating infection of developing B cells into the prevalent paradigm. Recurrent generation of virus-positive developing B cells may result from de novo infection by virus particles released from a BM resident cell type, de novo infection by virus particles released from reactivating plasma cells transiting to the BM, or a population of infected, self-renewing progenitor B cells. Virus-positive developing B cells subsequently transit through normal B cell differentiation pathways, resulting in constant replenishment of the infected memory B cell compartment. Figure was created with BioRender.com.

Defining the roles for developing B cells in chronic gammaherpesvirus infection is an enormous challenge. First and foremost, the vast complexity of the cellular composition and architecture of the BM, along with the low number of virus-infected cells, creates a tremendous barrier to identification of infected cell populations in the BM—similar to identifying the proverbial needle in the haystack. Although occasional “needles” can be located, proving that these rare cells are not just technical or biological artifacts is exceptionally difficult. However, emerging spatial imaging and transcriptomics technologies may greatly facilitate future studies. Regardless, the findings accumulated to date in EBV, KSHV, and MHV68 studies point to an unappreciated role for developing B cells in chronic gammaherpesvirus infection and disease. Based on this evidence, we hypothesize that gammaherpesviruses usurp natural B cell activation and maturation pathways to enable infected developing B cells to gain entry to the circulating mature B cell compartment [31,32]. Accordingly, we propose here a new working model that incorporates developing B cell infection data into the current textbook paradigm of gammaherpesvirus latency establishment in the B cell reservoir. The most prevalent model (**Fig 1, top panel**) holds that gammaherpesviruses initially infect naïve B cells and then, independent of antigen, drive those infected cells through a germinal center reaction and into the memory B cell compartment, which serves as a stable reservoir for lifelong latency. The new working model (**Fig 1, bottom panel**) expands this concept by incorporating developing B cell infection as a key contributor to both the establishment and maintenance of the latent B cell reservoir. In this scenario (which does not exclude initial simultaneous infection of naïve B cells): (a) Initial infection of developing B cells and expression of latency gene products leads to subsequent differentiation of cells and entry into the circulating mature B cell population, providing a conduit for establishment of latency in the B cell lineage; (b) Recurrent generation of virus-positive developing B cells may result from de novo infection by virus particles released from a BM resident cell type, de novo infection by virus particles released from reactivating plasma cells transiting to the BM, or differentiation from a population of latently infected, self-renewing progenitor B cells. Subsequently, infected developing B cells would presumably transit through normal B cell differentiation pathways, resulting in constant, low-level replenishment of the infected memory B cell compartment.

Future work will be required to systemically define virus and host determinants that mediate gammaherpesvirus infection of developing B cells and to directly address the specific contribution of developing B cell infection to the maintenance of long-term latency. Certainly, MHV68 infection of mice should allow the systematic investigation of each developing B cell subtype to determine their role in susceptibility of initial infection and latency and should provide a framework for new paradigms of the natural pathobiology of gammaherpesviruses in B cells. However, extensive additional work with human samples will be required to validate this working model and elucidate the consequence of developing B cell infections for human malignancies and autoimmune diseases.
